# Thyrotoxic periodic paralysis in a patient with Graves’ disease: A case report

**DOI:** 10.1016/j.amsu.2022.104447

**Published:** 2022-08-19

**Authors:** Sanjeev Kharel, Rajeev Ojha, Naresh Parajuli, Shiva Bhattarai, Gaurav Parajulee, Ragesh Karn, Bikram Prasad Gajurel, Reema Rajbhandari, Neeraj Gautam, Ashish Shrestha

**Affiliations:** a Maharajgunj Medical Campus, Tribhuvan University Institute of Medicine, Kathmandu, Nepal; b Department of Neurology, Tribhuvan University Teaching Hospital, Maharajgunj, Kathmandu, Nepal; c Department of Endocrinology, Tribhuvan University Teaching Hospital, Maharajgunj, Kathmandu, Nepal; d Janaki Medical College and Teaching Hospital, Janakpur, Nepal

**Keywords:** Thyrotoxicosis, Hypokalemia, Paralysis

## Abstract

**Introduction and importance:**

Thyrotoxic periodic paralysis (TPP) is a rare and often misdiagnosed, hypokalemic periodic paralysis with features of mainly recurrent acute limb weakness with good treatment outcome if diagnosed early.

**Case presentation:**

We here report a case of a 25-year-old male with a history of recurrent bilateral upper and lower limbs weakness resolved by potassium infusion later found to have Thyrotoxicosis (Graves’ disease). MRI scans of the brain had no abnormal finding while thyroid scintigraphy showed diffuse toxic goiter.

**Clinical discussion:**

Graves’ disease shares a majority of TPP while, other causes like toxic adenoma, thyroiditis, toxic multinodular goiter, amiodarone induced thyrotoxicosis, levothyroxine intoxication and thyrotropin (TSH) producing pituitary adenoma are also associated with TPP. The management of thyrotoxicosis by medical therapy, surgery or radioactive iodine therapy is the mainstay of treatment of TPP patients. For the treatment of acute attacks, potassium administration is necessary keeping in mind the problem of hyperkalemia because of excess doses of potassium as it shifts to extracellular space.

**Conclusion:**

TPP should be considered as a differential in the cases of limb weakness and the secondary causes especially Thyrotoxicosis and precipitating factors should be identified.

## Highlights

• Thyrotoxic periodic paralysis (TPP), a type of hypokalemic periodic paralysis, is either primary or secondary.
• The patients suffering from TPP have recurrent attacks of reversible muscle weakness and hypokalemia.
• TPP should be considered as a differential in the cases of limb weakness and the secondary causes especially Thyrotoxicosis.

## 1 Introduction

Thyrotoxic periodic paralysis (TPP), a type of hypokalemic periodic paralysis, is either primary or secondary. The primary is of familial type while the causes for secondary are thyrotoxicosis, hyperaldosteronism, nephritic syndrome, diabetic ketoacidosis, drugs, diuretic or laxative abuse, vomiting and diarrhea [1]. TPP and Familial Hypokalemic Periodic Paralysis(FHPP) have similar clinical features, but have different treatment strategies [2]. The patients suffering from TPP have recurrent attacks of reversible muscle weakness and hypokalemia [3]. Due to its rarity and lack of awareness, it is often misdiagnosed or delay diagnosis [4]. We here report a case of a 25 year old male with a history of recurrent bilateral upper and lower limbs weakness resolved by potassium infusion later found to have Thyrotoxicosis(Graves’ disease).

Our case has been reported in line with SCARE criteria [5].

## 2 Case presentation

A 25-year-old male bakery worker by occupation, presented to the outpatient department (OPD) of Tribhuvan University Teaching Hospital with a six weeks history of episodic weakness over bilateral upper and lower limbs. He initially noted numbness in his feet, followed by difficulties with walking, standing and unable to bear weight. A total of three similar episodes occurred during last six weeks. Two episodes were improved with potassium infusion in 7–8 hours in the local hospital but following third episode, the patient landed up in our OPD. There was no history of fever, headache, vomiting, trauma, loss of consciousness, trouble swallowing, weakness or difficulty in balance, speech and vision. The patient denied any loss of sensation, autonomic dysfunction, or changes in bowel or bladder habit. The patient didn't have a history of palpitation, weakness, weight loss, heat intolerance and anxiety. He did not give a history of surgery, medicine intake, vaccination, or exposure to toxins/heavy metals. The patient consumes a non-veg diet. The patient is an occasional drinker and chronic smoker with 1.5 pack years. There is no family history of similar complaints or any known neurological disease.

On admission, his general condition was fair with Glasgow coma score of 15/15. His vital signs were stable and there was no pallor, icterus, lymphadenopathy, edema, cyanosis, or clubbing. On neurological examination, his higher mental functions, cranial nerves, sensory and coordination tests were normal with no meningeal signs. Motor examination showed bilaterally symmetrical normal muscle bulk, normal tone, normal power (5/5) in the major joints and all groups of muscles of both upper and lower limbs and reflexes were also normal. The rest of the systemic examination findings were normal.

His hematological profile showed a normal blood count. His serum urea and creatinine levels were normal, sodium was 135 mEq/l and potassium 2.6 mEq/l. His fT3 level was 46 pmol/L(Normal range: 4.6–9.7 pmol/L), fT4 level 34.66 pmol/L(Normal Range:12–30 pmol/L), thyroxine stimulating hormone level 0.0013 **μ**IU/mL (Normal Range:0.4–4.5 **μ**IU/mL), serum ACTH levels 32.30pg/mL (Normal Range:10–60 pg/mL) and 3-am cortisol levels 14.60 **μg/dL(Normal Range:5–25 μg/dL)**

MRI scans of the brain had no abnormal finding. 99 m T_c_ Thyroid scintigraphy was done which showed diffuse toxic goiter. Based on symptoms, lab parameters and scintigraphy, patient was diagnosed as Graves’ disease (Thyrotoxicosis).

Hence, the presence of episodic muscle weakness along with hypokalemia and resolving of symptoms after potassium infusion in the background of Graves' disease (Thyrotoxicosis) while simultaneously ruling out other differentials pointed towards the diagnosis of Thyrotoxicosis periodic paralysis secondary to Graves’ disease.

During the course of hospital stay, the patient was treated in lines of thyrotoxicosis and hypokalemia. Acetazolamide, Carbimazole and propranolol were administered. The patient showed signs of improvement in his weakness. Patient's muscles power was completely recovered during discharged and was advised to continue the above stated medicines and intake of potassium chloride syrup if weakness reappeared. He was also advised to avoid high carbohydrate intake and heavy work. In follow-up after 6 weeks, patient was doing perfectly fine with no episodes of weakness after discharge.

## 3 Discussion

Thyrotoxicosis periodic paralysis is a rare and potentially life-threatening disease. Hyperthyroidism, hyperaldosteronism, diabetic ketoacidosis, nephrotic syndrome, drugs, acute tubular necrosis, laxative or diuretic abuse, diarrhea and vomiting are the secondary causes for the condition [2]. Though, Graves' disease shares a majority of TPP other causes like toxic adenoma, thyroiditis, toxic multinodular goiter, amiodarone induced thyrotoxicosis, levothyroxine intoxication and thyrotropin (TSH) producing pituitary adenoma are also associated with TPP [4]. Though there is a higher incidence of thyrotoxicosis in females, male to female ratio for TPP is 20:1. In Western countries FHPP is seen most common while in Asian TPP is most frequent [6]. TPP is seen most among males of aged 20–40 years. Reports of occurrence in children and adolescents are also found [4]. Our case is of a 25-year-old male Asian patient who was later diagnosed to have Graves’ disease (Thyrotoxicosis).

In TPP, patients experience acute weakness to complete paralysis and the episodes last from a few hours to 3 days. The sudden onset of weakness is a characteristic feature of TPP. The lower extremities are affected more than upper extremities while proximal muscles are affected more than distal muscles. About 80% cases of TPP have all four extremities involved. The degree of hypokalemia is directly correlated with severity of weakness. TPP was also known as “nocturnal palsy” as most attacks were in nights. There are many precipitating factors for recurrent paralytic episodes like heavy meals, alcohol, exercise, high salt diet, stress, infections, menstruation and glucocorticoids [2,4]. In our case, bilateral limb weakness was seen. Alcohol intake in our patient can be considered as one of the precipitating factors for recurrence.

The pathogenesis lies in the stimulation of Na+/K + ATPase activity by conditions like hyperthyroidism, hyperinsulinemia, and androgen access [7]. Guillain-Barre Syndrome, Transverse myelitis and acute spinal cord compression are the differentials for TPP. One differentiating point is the normal bowel and bladder function [8]. Our patient had a normal bowel and bladder habit.

TPP patients usually have attacks in hyperthyroid state. Generally, there is elevation of serum thyroxine(T4) and low thyrotropin levels (TSH). While elevated T3 and normal T4 were found in studies mainly in patients of adenoma or Graves’ disease having T3 thyrotoxicosis [9]. TPP from FHPP can be differentiated through a sensitive and specific test Urine calcium to phosphate ratio>1.7 [10]. High fT3,fT4 and low TSH levels were found in our case. The significant ECG changes seen in TPP patients is a triad of prolonged QT-U interval, prolonged PR interval, and a resting sinus tachycardia. Excess intravenous potassium, dextrose infusion, adrenaline administration for correction of hypokalemia and paralysis can precipitate ventricular arrhythmias through further decrease of potassium levels [11]. Our patient had a normal ECG finding when done at our center.

The management of thyrotoxicosis by medical therapy, surgery or radioactive iodine therapy is the mainstay of treatment of TPP patients. For the treatment of acute attacks, potassium administration is necessary keeping in mind the problem of hyperkalemia because of excess doses of potassium as it shifts to extracellular space [12]. Our patient was also given potassium infusion at the initial weakness event. There was no correlation seen between potassium dose administered and recovery time. β-adrenergic blocker like propranolol can be administered to prevent attacks until euthyroid state is achieved. Low-carbohydrate diet and potassium-sparing diuretics are other preventive measures [13]. The potassium supplements should not be given to patients between episodes as it is not useful for prophylaxis against further attacks [8].

Propranolol administration interferes with Na/K-ATPase channel can decrease thyrotoxic symptoms, raises serum levels of potassium and phosphate and also can address the problem of rebound hyperkalemia [8]. Study showed that use of antithyroid medications alone resulted in relapse attacks or recurrences among 56% of patients in one to seven months [14]. Strict medical compliance is related to prevention of recurrent paralytic episodes. The precipitating factors like heavy carbohydrate meals, high-salt diet, alcohol use, and significant physical activity should also be avoided by patients [8]. The above treatment modalities were provided to our patient causing him an early recovery and free of episodes of weakness.

## 4 Conclusion

TPP is a rare disease often misdiagnosed or difficult to differentiate from FPP. TPP should be considered as a differential in the cases of limb weakness and the secondary causes especially Thyrotoxicosis and precipitating factors should be identified. Physicians should be aware of progression of the disease and its appropriate management.

## Author agreement statement

We the undersigned declare that this manuscript is original, has not been published before and is not currently being considered for publication elsewhere.

We confirm that the manuscript has been read and approved by all named authors and that there are no other persons who satisfied the criteria for authorship but are not listed. We further confirm that the order of authors listed in the manuscript has been approved by all of us.

We understand that the Corresponding Author is the sole contact for the Editorial process. He/she is responsible for communicating with the other authors about progress, submissions of revisions and final approval of proofs.

## Ethical approval

This is a case report, therefore, it did not require ethical approval from the ethics committee.

## Source of funding

The study did not receive any grant from funding agencies in the public, commercial or not-for-profit sectors.

## Authors contribution

RO, NP, NG and AS: involved in counselling and treatment of the patient. SK, RO and SB: collected all the required case information, images, reports; reviewed the literature and contributed in both writing and editing the manuscript. BPG, RG, RR and RP: involved in editing the manuscript. All authors read and approved the final manuscript.

## Registration of research studies

1. Name of the registry: Not applicable
2. Unique Identifying number or registration ID:
3. Hyperlink to your specific registration (must be publicly accessible and will be checked):

## Guarantor

Sanjeev Kharel.

## Consent

Written informed consent was obtained from the patient for publication of this case report. A copy of the written consent is available for review by the editor-in-chief of this journal on request.

## Provenance and peer review

Not commissioned, externally peer-reviewed.

## Declaration of competing interest

None.
